# Bilateral giant Becker's nevus^⋆^

**DOI:** 10.1016/j.abd.2021.05.024

**Published:** 2023-01-23

**Authors:** Li-Wen Zhang, Cun-Huo Jiang, Lin Li, Tao Chen

**Affiliations:** Department of Dermatovenereology, Chengdu Second People’s Hospital, Chengdu, Sichuan, China

Dear Editor,

A 13-year-old boy presented with a 6-year history of multiple asymptomatic brown macules affecting the chest and scapular regions bilaterally. The pigmentation gradually darkened and progressively involved the neck, right forearm, both shoulders, upper arms, and axillae (Figure 1, Figure 2). Increased hairs were observed on the lesions . No other accompanied abnormality of the skin or musculoskeletal system was found. Familial and medical histories were unremarkable. Laboratory investigations including complete blood cell count, liver, and renal function were all normal. Radiography of the chest, spine and upper extremities also showed no abnormality. The histopathological examination revealed slight hyperkeratosis, with acanthosis, elongation, and fusion of the epidermal ridges and hyperpigmentation of the basal layer (Fig. 3). These features were suggestive of Becker’s nevus.

**Figure 1:**
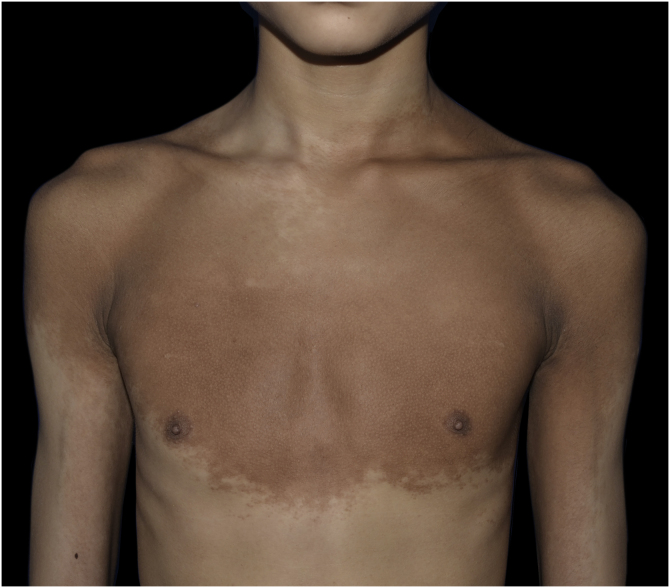
Multiple brown macules with hypertrichosis on the chest, shoulders, upper arms and axillae.

**Figure 2:**
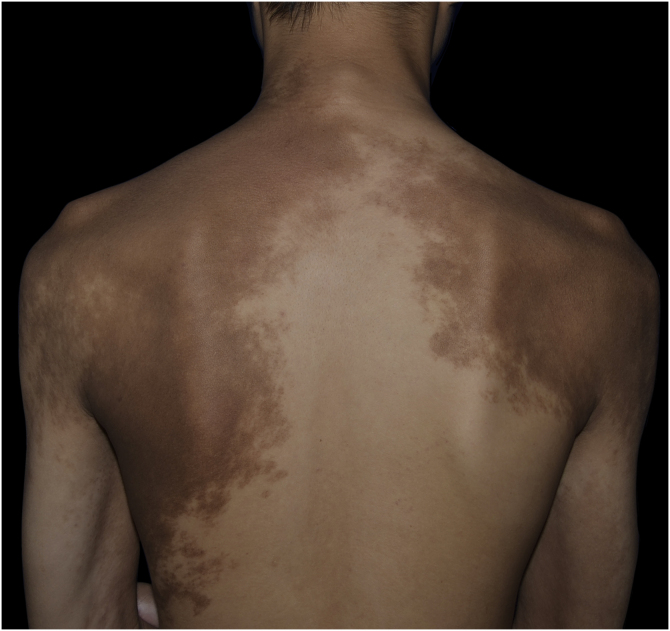
The pigmentation on the neck and scapular regions.

**Figure 3:**
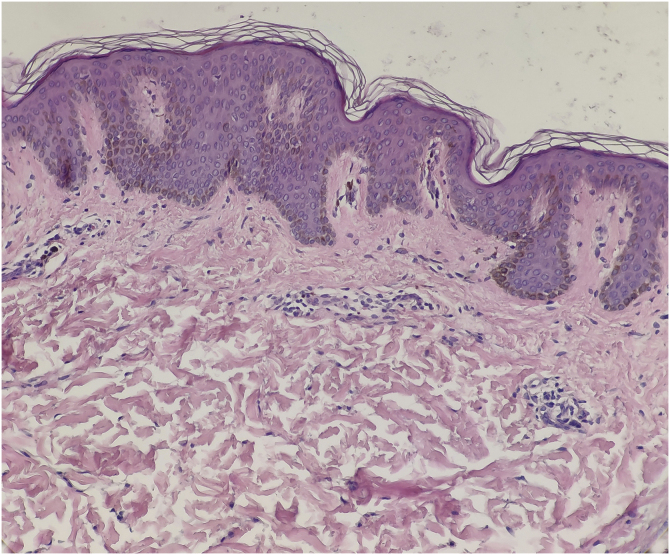
The histopathological examination revealed slight hyperkeratosis, acanthosis, elongation, and fusion of the epidermal ridges, with hyperpigmentation of the basal layer (Hematoxylin & eosin, ×200).

Becker’s nevus is a type of epidermal nevus, characterized by a single hyperpigmented patch with or without hypertrichosis, and predominantly involves the unilateral upper trunk, scapular region, or upper arm unilaterally, the disorder most likely reflects mosaicism. Becker’s nevus is an androgen-dependent lesion because it becomes more prominent after adolescence and tends to be more conspicuous in male patients because of increased hairiness of this area. The male-to-female ratio was said to be 2:1∼5:1.^1^, ^2^ However, some authors believed that the true sex ratio may be 1:1, due to Becker’s nevus tends to be less conspicuous in female patients.^1^, ^3^ The other pathogenetic hypothesis of Becker’s nevus was postzygotic mutations in beta-actin.^4^

Multiple or bilateral Becker’s nevus is rarely reported in the literature. We reviewed a total of 25 reported cases of bilateral Becker’s nevus recently.^5^ Among them, the male-to-female ratio was 18:7; 10 cases presented a single giant coalescent lesion; 15 cases were multiple separated lesions. The lesion distribution presented as symmetrical or asymmetrical (including 4 cases of checkerboard pattern). Like common Becker’s nevus, the majority of bilateral Becker’s nevus manifested in adolescence, and only a few patients present at birth or shortly after birth. There were 6 cases with extracutaneous abnormalities, so-called Becker’s nevus syndrome, including breast hypoplasia, musculoskeletal defects, mental retardation, and cardiac defects. The incidence of Becker’s nevus syndrome appears to be higher in bilateral Becker’s nevus. Therefore, patients with a diagnosis of bilateral Becker’s nevus should undergo clinical evaluation for extracutaneous involvement.

## Financial support

None declared.

## Authors’ contributions

Li-Wen Zhang and Cun-Huo Jiang contributed equally to this work. Li-Wen Zhang: Study conception and planning, preparation and writing of the manuscript. Cun-Huo Jiang: Acquisition of data. Lin Li: Literature review. Tao Chen: Approval of the final version of the manuscript.

## Conflicts of interest

None declared.
